# The Development of a Vocational Rehabilitation Program to Assist Individuals With MDRTB and TB in Returning to Work

**DOI:** 10.1155/oti/9914578

**Published:** 2025-01-04

**Authors:** Mogammad Shaheed Soeker, Ayesha Jainodien

**Affiliations:** Occupational Therapy Department, University of the Western Cape, Cape Town, South Africa

**Keywords:** adaptations, client-centred approach, occupational therapy, perception, qualitative research, return to work, self-efficacy, tuberculosis, vocational rehabilitation, young adults

## Abstract

**Background:** Individuals diagnosed with tuberculosis (TB) and multidrug-resistant (MDR) TB may struggle to return to work after they have completed a rehabilitation program. Multidrug-resistant tuberculosis (MDRTB) has been seen as a condition that is resistant to treatment, hence causing individuals to be economically in-active for considerable periods of time.

**Objective:** The aim of the current study was to explore the views of individuals living with MDRTB, individuals with TB, and health professionals treating individuals with TB and MDRTB about the development of a vocational rehabilitation program.

**Method:** The researchers used an exploratory descriptive research design, and semistructured interviews were conducted with five key informants and four participants who were diagnosed with pulmonary tuberculosis (PTB) and MDRTB. Thematic analysis was used in order to analyse the study findings. The current study is the second of two articles. The first article focused on barriers and facilitators linked to returning to work for individuals living with TB and MDRTB. The current article focuses on the development of a vocational rehabilitation program.

**Results:** The findings of the original study revealed five themes; however, for the purpose of this article, only two themes will be presented, namely, Theme 1: promoting a holistic model and Theme 2: the use of resources for activity engagement. The latter theme contributed to the participant's view of the development of a vocational rehabilitation program.

**Conclusion:** The study provided a description of the components of a vocational rehabilitation program that has been adapted from the Model of Occupational Self-Efficacy (MOOSE). The above program has been designed for individuals diagnosed with PTB/MDRTB and has the potential to assist them in returning to work. It is suggested that vocational rehabilitation programs be incorporated into general medical programs that focus on improving the functioning of individuals diagnosed with PTB/MDRTB.

## 1. Introduction

### 1.1. Epidemiology of Tuberculosis (TB)

TB has been seen as a devastating disease that has affected millions of individuals throughout the world and has been viewed as a public health threat. The World Health Organization (WHO) reported that in 2024, 2.5 million people became sick with TB in Africa, thus contributing to a quarter of new TB cases worldwide. WHO reported that in 2022, about 424,000 people died from the disease in the African region (1.267 million globally) [1]. Over 33% of TB deaths occur in the African region. TB has been regarded as one of the 10 leading causes of death in developing countries. Furthermore, according to WHO [2], it is reported that the risk of HIV positive people developing TB is about 40% more when compared to HIV negative people, with many individuals diagnosed with HIV residing in African countries. The WHO reported that 1.25 million people died from TB in 2023 (including 161,000 people with HIV), thus confirming TB as the leading cause of death from a single infectious agent [1].

The cases of TB incidence are still very high in developing countries such as South Africa [3]. Although South Africa has made good strides in decreasing the mortality related to the disease, the incidence of TB in South Africa remains at 468 per 100,000 of the population [3]. TB is the leading cause of death among young adults who are in the most productive years of their lives [4].

On average, 47.3% of individuals diagnosed with TB lose out on work productivity while they are recovering [4]. The above may have an economic impact on communities to the extent that up to a third of individuals who complete TB treatment are still unemployed [5]. Some of the common symptoms related to TB that influence an individual diagnosed with TB's ability to return to work are low physical endurance, loss of motivation, and a loss of self-esteem [6]. Other psychosocial factors include the stigma that society has towards individuals with TB due to the fear of becoming infected by them [7]. The worker roles of individuals diagnosed with TB are affected, as they often struggle to complete work tasks due to their symptoms of low physical endurance [5]. It could be argued that due to the unemployment rate, individuals with TB could experience stigma in the workplace as they may not be accommodated in the workplace. This may result in these individuals experiencing low self-esteem and negatively affecting their ability to hold jobs when becoming reinfected [5]. Many of these individuals may have low self-esteem and could struggle to maintain work when they get reinfected. Society may stigmatise individuals with TB and in particular multidrug-resistant tuberculosis (MDRTB), thus reinforcing low self-esteem among individuals diagnosed with TB [8, 9].

Rehabilitation programs such as work-hardening programs and supported employment programs have been commonly used in order to enhance the work skills of persons with disabilities [10]. However, it could be argued that there are minimal to no vocational rehabilitation programs that specifically focus on enhancing the work skills of individuals with pulmonary tuberculosis (PTB) and MDRTB in South Africa and other countries. The Model of Occupational Self-Efficacy (MOOSE) has been used with great success in South Africa, especially in enhancing the work skills and self-efficacy beliefs of individuals with disabilities [11]. Soeker, Abbas, and Karachi [12] have applied the rehabilitation model in different contexts and with individuals with diagnoses ranging from traumatic brain injury, stroke, and schizophrenia. Therefore, as a result of the poor return to work rates of individuals with TB and the fact that individuals with PTB and MDRTB may have reduced self-efficacy beliefs, the authors explored ways of adapting the MOOSE in order to enhance the work skills of individuals living with PTB and MDRTB.

### 1.2. Description of the MOOSE

The MOOSE is a vocational rehabilitation model that consists of four stages that are conducted by the occupational therapist treating the individual diagnosed with PTB and/or MDRTB. Stage 1 is termed *a strong belief in functional ability.* Self-reflection is the goal of this stage of the model; the individual is allowed to self-reflect on the incident (this could be the cause of the illness), therefore creating an opportunity for introspection. The process of introspection allows the individual to recognise and actively manage any emotions related to their circumstances [13]. Stage 2 is called the *use of self*; during this stage, the individual takes control of their life and develops a plan to overcome the difficulties they experienced with the assistance of the occupational therapist. The purpose of rehabilitation is to enhance their functional skills such as cognition and endurance [13]. Stage 3 is called the *creation of competency through occupational engagement*; during this stage, the individual's (patient) perception of their worker role changes from a worker needing assistance to a more positive independent worker role. This stage focuses on enhancing the individual's work-related skills, such as computer literacy, driving skills, and communication skills. This subsequently improves their work performance [13]. Stage 4 of the model is called *capable individual.* This stage is characterised by an improvement in the individual's volition and their worker role to the extent that they can successfully engage in work tasks, which then leads to an improved sense of self-efficacy. In this stage, the individual will be involved in test placements where they can actually practice their skills in real work settings. During the last stage of the model, the occupational therapist gradually reduces the amount of support provided, as the aim is to enable the individual who is receiving treatment to function independently [13].

**Figure 1:**
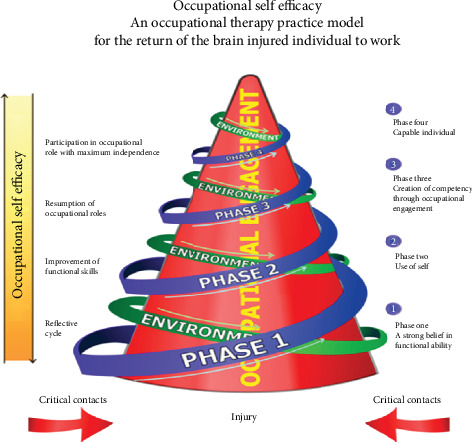
Model of Occupational Self-Efficacy.

In the current study, the MOOSE was adapted after the interviews were conducted with the research participants. The interviews were therefore used in order to adapt the MOOSE in order to enhance the work skills of individuals diagnosed with PTB and MDRTB. The steps of the MOOSE are graphically described in Figure 1.

### 1.3. Aim

The aim of the current study was to explore the views of individuals living with MDRTB, individuals with TB, and health professionals treating individuals with TB and MDRTB about the development of a vocational rehabilitation program adapted from the MOOSE.

## 2. Methods

A qualitative exploratory descriptive research design was used in order to describe the experiences of individuals with PTB/MDRTB and key informants (i.e., occupational therapists and physiotherapists) about their views of enhancing rehabilitation and in particular in adapting the MOOSE as a viable vocational rehabilitation program that would aid individuals with PTB/MDRTB in returning to work. Purposive sampling was used in order to select the participants. Nine participants were selected to participate in the study [14]. The number of research participants is based on the need to obtain detailed qualitative data on the usefulness of the MOOSE in treating individuals with PTB. According to Creswell [15], a sample size of between five and 25 is regarded as appropriate for qualitative studies, as the purpose of qualitative studies is to obtain an in-depth understanding of phenomena. The sample of nine individuals was therefore regarded as appropriate for the study. The purpose of the study was to understand the experiences of individuals with PTB/MDRTB and health professionals involved in the treatment of PTB and MDRTB about returning to work after they have completed rehabilitation.

## 3. Participants

The key informants consisted of five rehabilitation specialists using the following inclusion and exclusion criteria. For inclusion in this study, eligible participants had to be health rehabilitation experts such as occupational therapists or physiotherapists who have worked with PTB or MDRTB clients for at least 6 months and were involved or experienced in getting PTB and MDRTB clients back to work.

The second group of participants for this study consisted of four individuals living with PTB and MDRTB who have returned to work after completing rehabilitation at the TB/MDRTB specialist hospital. Individuals were included in the study if they were diagnosed with PTB and MDRTB. Individuals must have been employed before participating in the rehabilitation program at the TB/MDRTB specialist hospital.

## 4. Procedure

The research participants were selected by viewing the hospital records of individuals diagnosed with PTB and MDRTB held at hospitals that specialize in treating individuals with PTB/MDRTB. The researchers used one hospital in Cape Town that specializes in the treatment of individuals diagnosed with PTB and/or MDRTB. Through a telephone call, the participants were given brief information about the study, and the researcher ensured that the participants met the criteria for inclusion and then arranged an appointment to discuss possible participation and interest. Once individuals indicated that they were interested in the study, they were requested to provide informed consent. All of the introductory meetings and interviews took place in the occupational therapy departments at the hospitals.

## 5. Data Collection

Two individual semistructured interviews were completed with each one of the five key informants and four participants about returning to work after they had completed their rehabilitation. The researcher used a semistructured interview guide, with open-ended questions focusing on the barriers or challenges that the research participants experienced when using MOOSE. Other open-ended questions focused on the facilitatory factors or positive factors of the MOOSE that helped individuals with PTB and MDRTB enhance their work skills. Finally, the last open-ended question focused on the views of the research participants about what changes could be made to the MOOSE in order to enhance its usefulness in order to improve the work skills of individuals diagnosed with PTB and/or MDRTB.

## 6. The Process Followed to Adapt the MOOSE in Order to Treat Individuals With TB/MDRTB

The program that used MOOSE as a framework was adapted by means of reviewing the literature related to individuals with TB/MDRTB, the perspectives of both individuals with TB/MDRTB, and key informants about adapting MOOSE as a program to treat individuals with TB/MDRTB. The proposed adaptations to the MOOSE were described, and its layout is in a table format (this can be viewed in Table 1). For the purpose of this article, Table 1, which is the outcome of the study, will be presented in the results section of this article.

## 7. Analysis and Trustworthiness

In this study, the eight steps of Tesch's [16] qualitative data analysis methods were used in order to formulate themes. In the first step, the researcher reads the interview transcripts and documents ideas of significance related to the study. In Step 2, similar topics or meanings related to the study were extracted and grouped together. In Steps 3–7, descriptive codes were formed that eventually resulted in the subcategories and categories being formed. In Step 8, the finalized themes were formed and presented. The trustworthiness of the qualitative study was formed by the researchers using the strategies advocated by Krefting [17], namely, four basic criteria, namely, truth value, applicability, consistency, and neutrality of data.

## 8. Ethics

The guidelines advocated by WHO [18] were used in order to promote ethics related to research by foregrounding the rights of the research participants. The participants were provided with a document describing the aim of the study, particularly concepts such as informed consent, anonymity, confidentiality, and nonmaleficence.

## 9. Results

The participants described the various factors that contributed to the development of a holistic model of rehabilitation. Their ages ranged between 39 and 56 years. Only one participant was under 40 years old. Four participants were between the ages of 40 and 50, five between the ages of 50 and 60, and none above 60 years of age. Seven of the participants were female, and two were male [14]. Participant demographic data are provided in Table 2 (see Table 2).

Five themes emerged from the study, but for the purpose of this article, only two themes will be described. The two themes below focus mainly on the views of the research participants with reference to recommendations related to adapting the MOOSE for individuals diagnosed with PTB and/or MDRTB.

Two themes were described, namely, Theme 1: promoting a holistic model and Theme 2: the use of resources for activity engagement. The latter theme contributed to the participant's view of the development of a vocational rehabilitation program.  Table 3 describes the results of the study.

### 9.1. Theme 1: Promoting a Holistic Model

The above theme represents the participants' comments on the adapted program. The rehabilitation specialists reflected that the adapted program focused on various areas impacting the lives of TB and MDRTB patients and could therefore be considered a holistic approach to facilitating return to work for this target group. The participants also indicated that the adaptation assisted with community integration and return to work as a rehabilitation intervention. The quote below illustrates this theme:

So the program itself like I said, is a holistic program … like prevocational skills training is one component of it,…it includes like, life skills, education groups, support groups, uhm just leisure, sport and leisure, and recreation, arts and crafts and then substance abuse. (P1: rehab specialist)

Another participant indicated that the adapted program recognizes the type of clients that will be engaging in the rehabilitation program and their contextual reality. She said:

…when I say realistic, [it looks] at the context where they coming from because some of them…some of them don't have the resources, uhm you know the resources to achieve those goals, so we look at the context where they coming from, the community…where they are going back uhm so we try and make it as realistic as possible… (P4: rehab specialist)

Theme 1 consisted of two categories, namely, developing a client-specific program and therapist involvement.

#### 9.1.1. Developing a Client-Specific Program Based on Context

This category describes the focal point of the program. Most of the participants felt that the adapted program appeared to be guided by the needs of the clients/patients. One participant explained that the tasks and activities proposed for the client/patient to engage in are meaningful, which in turn would benefit the client/patient. He said:

tasks [that] they can't actually do [it] outside [is] not really meaningful. So I think those [are the] kinds of things to consider uhm when drawing up (program). (P1: rehab specialist)

This was further captured by another participant, who stated:

…programs [must be based] on what the patients' needs are and what they say they're interested in, if they don't come up with stuff then we would recommend certain things. (P2: rehab specialist)

The participants identified a number of features of the adapted MOOSE which is an example of holistic programming.

In addition to the program being context-specific, the research participants advocated the need for TB survivors to receive early intervention. One of the participants indicated:

Equip them just to make sure they understand that it's not the end of road if you have the disease. It's just, you must make sure that you are going to finish your treatment. (TB2: TB survivor)

#### 9.1.2. Therapist Engagement in the Program

This category is representative of the participant's view of the extent of therapist involvement in the activities included in the adapted program. The category is described by two subcategories, namely, Subcategory 1—understanding the client's context—and Subcategory 2—the therapist as an external support and motivation.

Activities such as curriculum vitae writing and group work are labour-intensive. Participants expressed that the adapted program may not be feasible given the limitations of the public health care system. The participants identified that group work is adopted as a compromise to ensure that therapists can be involved with more clients at the expense of depth work at an individual contact level. Individuals who have been attending group Occupational Therapy (OT) programs felt that it contributed to their individual goals as well. The participant said:

I used to do that and then each and every day even on Sundays I have to go to OT (group OT program) and then doing the program, for me it was fine and good because it made me feel comfortable and then believed in myself. (TB2: TB survivor)

##### 9.1.2.1. Understanding the Client's Context

The participants underscored the importance of understanding the population of patients that are admitted for PTB and MDRTB treatment. The ability of therapists to facilitate return to work is predicated on the extent to which the target group has a premorbid appreciation of employment and functional work. The participants reflected that the PTB and MDRTB patients might not have been employed prior to being admitted to the hospital. A participant stated that some clients/patients would need a lot of education to understand the worker's role. Thus, in reviewing the adapted program, participants identified that the inclusion of work-related activities was contextually sensitive and relevant for the target group. Another participant described this by stating:

I was very emotional and I was very depressed and so at the progress he helped me to overcome it and I do a lot of things here like I learn to work on a sewing machine and I show the other patients what to do and work in groups and I'm the one who show them what to do and how to do it because needlework was my thing that I do yeah. (TB3: TB survivor)

##### 9.1.2.2. Therapist as External Support and Motivation

The participants reported that the adapted program did not specifically speak to the role of therapists in providing support and motivation to patients. The adapted program provided appropriate activities for engagement with patients but did not structurally provide for the explicit provision of support and motivation to patients, given that patients have PTB and MDRTB. In the adapted program, the therapists should focus on enhancing the individual with TB/MDRTB's motivation to believe in themselves to engage in their worker role.

One participant indicated this by stating:

I used to do that and then each and every day even on Sundays I have to go to OT and then doing the program, for me it was fine and good because it made me feel comfortable and then believed in myself. (TB2: TB survivor)

### 9.2. Theme 2: The Use of Resources for Activity Engagement

Theme 2 addressed the resources for activity engagement in the adapted program for patients with PTB and MDRTB. The theme included two categories, namely, (1) resources and a need for staff in public health care and (2) vocational rehabilitation assessment tools.

#### 9.2.1. Resources and a Need for Staff in Public Health Care

In this category, participants underscored the importance of considering the resources available in rehabilitation settings where the adapted program would be implemented. They identified that the lack of physical, human, and financial resources would pose a threat to the resumption of the survivor's worker role. Most participants were of the opinion that improved services to the PTB and MDRTB survivors could be delivered if they had the necessary resources. One participant captured this description by stating:

Here at DP (name of hospital) we don't really have an existing program officially for people who need to or who would want to return to work because uhm we don't have the you know…the necessary uhm how can I say…materials or necessary equipment for that. (P4: rehab specialist)

Another participant described this by stating:

… we're very limited with resources, human and physical resources. (P2: rehab specialist)

These quotes illustrate the context within which the adapted program would be used to facilitate return to work for the targeted clinical group. This reality makes it necessary to work collaboratively in order to manage the lack of resources.

If we merged more often (work collaboratively) surrounding our patients then it would definitely work a lot better and the patient will have a better outcome (P3: rehab specialist)

The participants felt that the adapted program lends itself to collaborative service delivery. The activities included may be labour-intensive, but the way in which it is compiled allows rehabilitation specialists to work together if there are human resource constraints. Collaboration made possible by the interprofessional nature of the activities included in the adapted program could be more of a benefit to the PTB and MDRTB survivors. This category has two subcategories. Below is a brief explanation of these subcategories.

##### 9.2.1.1. The Lack of Resources Affects the Quality of Treatment Provided

This subcategory conveys the participants' concerns that delivering good-quality rehabilitation or intervention descriptions is contingent on sufficient resources. Thus, the adapted program should be robust enough to maintain its good quality despite the lack of resources such as a lack of experience of therapists in working with individuals with TB/MDRTB. The participants were of the view that maintaining the quality of the OT vocational rehab program was helpful in getting them to achieve their individual goals. One participant said:

Yes, I did achieve many goals because I did set the goals, I remember the time I was admitted there I said: ‘I'm going to achieve the goals' and then I said: ‘No the first week I must finish this, I must do that' and now I finish this, and this and then I start the other one. (TB2: TB survivor)

##### 9.2.1.2. Skill Development

Participants in this study were of the opinion that engagement in work skills was not focused on enough during rehabilitation, as well as the promotion of new work skills. They indicated that therapists often overlooked vocational rehabilitation in favour of the physical rehabilitation of the client/patient. Participants felt that the adapted MOOSE specified activities that promoted skills development during the rehabilitative phase. The above subcategory indicates that rehabilitation services need to focus more on skill development that promotes vocational rehabilitation. One participant said:

It helped me a lot and you know what, I'm not at work at the moment but I do lot of things (work related products to sell) and I sell it and to the people. (TB3: TB survivor)

#### 9.2.2. Lack of Vocational Rehabilitation Assessment Tools

This category represents the participants' perceptions of the lack of vocational rehabilitation assessment tools that are made available in government-run health institutions. Participants underscored that work skills are often sacrificed when there are financial constraints that impact material resources. The category is further described with subcategories relating to participants' experiences and perceptions of vocational rehab standardized assessments and engagement in vocational rehabilitation.

##### 9.2.2.1. Usefulness of Vocational Rehab Standardized Assessments

This subcategory conveys the description of the usefulness of vocational rehabilitation standardised assessments. Participants in this study were of the opinion that standardised assessments would provide more of an accurate representation of the client/patient's work ability. She described this by stating:

Looking at also getting equipment, and more standardized tests where the work assessment, the result could be more accurate. (P4: rehab specialist)

The participant further emphasized this point by saying:

The doctor will sometimes refer us, refer the patients to us for a disability grant scheme, uhm but it's not how can I say… It's very basic because we don't like I said have the necessary equipment like the Valpar and Modapts or all of those things to actually assess them in the way that they supposed to be assessed… (P4: rehab specialist)

##### 9.2.2.2. Engagement in Vocational Rehabilitation

This subcategory addressed the extent to which the adapted program makes vocational rehabilitation tasks available for PTB patients to engage in. One participant expressed that it would be easier to encourage engagement in work-related tasks or activities given that it is included in the adapted program. The inclusion of vocation-related activities and skill development will increase patients' interest, which in turn will promote learning and engagement. One participant identified that these aspects of the adapted program will contribute to the sustainability of return to work and integration back into the community. One participant identified that the adapted program should make the link between the activities and the rehabilitation outcome clearer for patients and therapists.

OT historically is known for just keeping you know that whole “keeping people busy” the new shade term uhm but they do not see what the purpose of the interventions are. So there maybe there will be car washes and income generating activities, but the rest of the team they aware of what's happening but they do not understand the meaning behind the activities. (P1: rehab specialist)

The above quote describes that clients/participants need to understand why they are being taught new skills or engaging in specific work-related tasks. This insight or linkage between theory and practice was less apparent in traditional OT interventions.

### 9.3. Recommendations to Enhance the MOOSE

The adapted MOOSE program focuses on nine recommendations that create the framework of the program. (1) Recommendations were related to the use of various activities for enhancing the functional limitations of the individual diagnosed with TB/MDRTB. (2) There were recommendations related to the therapist's role; this describes the therapeutic approach to be used by the health professional. (3) There were amendments to the assessment methods used that linked to the stage of treatment. (4) There was information related to clinical outcome(s) linked to each stage of the model that was added. (5) Information related to the duration of the treatment session was added. (6) This was followed by the core constructs, illustrating what the therapist should focus on. (7) Information related to the suggested time frame and amount of treatment sessions was added. (8) Information related to the suggested treatment approach that health professionals should use was included. (9) Finally, information related to program structure was added [14].

The latter components serve as an overarching framework of the program that will enable the individual with TB/MDRTB to engage with treatment from the start of the program until the period when they complete the program. The original stages of the MOOSE were consistent with the adapted stages of MOOSE that were currently more suited to enhance the work skills of individuals with PTB/MDRTB (please see Table 1—adapted MOOSE).

## 10. Discussion

In the following section, the results that relate to the views of both participants and rehabilitation experts regarding the adapted MOOSE will be discussed. Themes 1 and 2, respectively, related to the participants' views of the adapted program.

### 10.1. Theme 1: Promoting a Holistic Model

Theme 1 “promoting a holistic model” was viewed as suggestions to adapt the content of the original MOOSE in creating the new adapted MOOSE. The study participants expressed that the rehabilitation program for patients with PTB and MDRTB should be one that is holistic and focuses on all aspects of improving one's health and well-being. Asbjørnslett, Skarpaas, and Stigen [20] explained that occupational therapy is based on the philosophical core principals of providing holistic and client-centred therapy [21]. Through the use of holistic therapy, individuals have better health for longer, and the older generations have more sense of hope that their end of life can be pleasurable and good [22]. Thus, both the adapted program and the implementing therapists must take into consideration the context that the patient comes from and the various areas that impact the lives of TB and MDRTB patients. Community reintegration, family support, prevocational skills, and work rehabilitation need to be prioritised as these areas are often overlooked due to the main focus being medical and physical rehabilitation.

#### 10.1.1. Developing a Client-Specific Program Based on Context

Participants of the current study felt that the adapted program was informed by the needs of the clients/patients. The needs of the client/patient thus guide which areas of treatment require the most attention and intervention, and subsequently which tasks or activities will be meaningful to the client/participant. This notion was consistent with Fisher and Martella [23], who explained meaningful occupation as being the engagement or participation in an activity, to the extent that a person values it. They stress the importance of understanding occupation as a transactional whole in which occupational and situational elements interlink. This view of the occupational therapy profession stems from evidence that was found through research, where participation in meaningful occupations, including work, generally leads to increased feelings of overall well-being [24]. This finding is consistent with Bigelius, Eklund, and Erlandsson [25], who stated that if occupations are not meaningful, then they cannot be therapeutic. Lastly, Ikiugu et al. [26] reported that researchers have come to the agreement that meaningful occupations provide people with a sense of control; it gives them an identity, allows for a connection with other people, and develops competence and self-expression. The adapted program was deemed suitable for clients/patients who come from various types of contexts so it is applicable to most people. Therefore, the types of tasks and activities included were ones that either relate to their previous worker role or ones that they have an interest in, for the potential to learn a new skill. Rehabilitation specialists must endeavour to fully understand the context where these clients/patients come from so that these patients can acquire skills that are achievable or apply for jobs that will be suitable for them. The adapted program retained a staged approach so that the client/patient can start off at a level that is suitable for him or her, where realistic goals can be set, and engage in appropriate activities that will be applicable to the level of work experience.

### 10.2. A Need for Resources Required for a Holistic Rehabilitation Program

Theme 2 describes the resources required in order to enable activity participation. It is important for public rehabilitation facilities to be equipped with enough staff to facilitate these programs, as well as have therapists who will be available to facilitate treatment for patients who have specific or individualised job roles. However, in this study, the participants relayed that there is a lack of staff in the public health sector. Therefore, most intervention programs are exclusively facilitated through group therapy, so that they are able to assist all patients. However, this is not always the best method of intervention, as every person has individualised needs, and this limits the person who comes from a different context and has different skill capabilities. When clients/patients learn new skills, the therapist will encourage and empower the patients to be able to utilize those skills to find new jobs once they reintegrate back into the community and explore new worker roles, or encourage them to resume their previous worker role provided [27].

#### 10.2.1. Lack of Vocational Rehabilitation Assessment Tools

The lack of vocational rehabilitation assessment tools specifically for the treatment of individuals with PTB and MDRTB was seen as a limitation in the current study. Tools in this study were seen as strategies to assess the functional limitations but more specifically the work-related skills of the clients/patients. The utilisation of standardised tests was seen as needs, example tests that could guide the therapist in identifying their work abilities such as their work endurance, ability to manage tools and equipment, ability to work with coworkers, and their general work speed. According to van Aswegen and Roos [28], they indicate that individuals with TB, in general, have low physical endurance, and it could therefore be argued that their ability to work an 8-h workday will be compromised. It is therefore essential to improve the client's work endurance with relevant work-related tasks. Furthermore, the ability to use work-related tools and communicate effectively with others by means of engaging in a simulated work environment is seen as important, particularly in enhancing their work skills.

## 11. Limitations to the Study

The recruitment of PTB survivors who returned to work was challenging. Only one male participant was recruited in the PTB and MDRTB survivors' subgroup. Despite concerted efforts to recruit from both genders, it was difficult to identify male participants. From the health professional's perspective who acted as key informants, it was difficult to recruit male participants as the professions of occupational therapy and physiotherapy are dominated by females. Finally, the small sample size could be seen as another limitation of the study.

## 12. Conclusion

The findings of the study described two themes, namely, “promoting a holistic model” and “the use of resources for activity engagement,” that provide information pertaining to adapting the MOOSE. The adapted MOOSE provides a detailed description of the use of a vocational rehabilitation program that could be incorporated as part of a medical program for the treatment of individuals with TB/MDRTB. The importance of OT services in TB rehabilitation emerged through the perception of the rehabilitation care specialists as they identified the lack of public rehabilitation services that focus on work rehabilitation. The revised program describes the importance of having individuals with TB/MDRTB access work-related intervention programs and engages in work test placements as part of a general rehabilitation program.

## Figures and Tables

**Table 1:** Adapted model of occupational self-efficacy.

**Activities**	**Therapist role**	**Assessment**	**Clinical outcomes**	**Session**	**Core constructs**	**Time frame**	**Treatment approach**	**Program structure**
Adapted Model of Occupational Self-Efficacy—Stage 1 Stage 1: Stage 1 will occur over a duration of 1 week								
Role play	A client-centred approach to treatment will be used. The client's insight into the diagnosis will be assessed.	Assessment of specific components (i.e., range of motion, muscle strength, sensation, conation, and cognition)	Stage 1: A strong belief in functional ability • This stage is aimed at introspection and self-reflection on the incident and the feelings surrounding their new life circumstances post-TBI. • The outcome of this phase is very similar to the first stage of the original model. Using the Gibbs reflective cycle, with focusing on introspection. The client reflects on the change that has occurred and needs to have insight into his/her diagnosis and accept his/her new capabilities and challenges with the goal of going back to the workplace.	60 min session	The client reflects on the physical, emotional, and psychological changes. Focusing on then and now. Telling the group, the problem, getting feedback from peers.	3 × 60 min sessions once a week	Group therapy	Components to improve: - Social skills - Arousal level - Cognitive behaviour skills - Creative skills - Reflection - Self-image
Simulated work activities		• Modapts • Valpar • Work samples		45 min session	Simulated work tasks that are specific to the client. Focus on work-hardening skills.	1 × 45 min session once a week	Individual therapy	Components to improve: - Endurance - Muscle strength - Range of motion - Self-image

Adapted Model of Occupational Self-Efficacy—Stage 2 Stage 2: Stage 2 will occur over a duration of 2 weeks								
Skill training—Microsoft Word	The therapist will facilitate basic training on Microsoft Word to those participants who fit the criteria.	- Work samples (e.g., Modapts and Valpar) - Assessment of specific components (i.e., range of motion, muscle strength, sensation, conation, and cognition) - Assessment of work abilities (i.e., work habits, work competence, and work endurance)	Stage 2: Use of self • During this stage, the client regains control of their life situation and realizes their potential. • During this stage, the participant should have insight into his/her coping strategies and enhance their sense of self-efficacy. • Patients should be competent in understanding the meaning of self-efficacy before entering the next stage.	60 min session	Focus on critical thinking skills and problem-solving. Encourage the client to find logical and practical solutions to problems.	5 × 60 min sessions for 1 week.	Group therapy.	Components to improve: - Memory - Sequencing - Attention span - Concept formation - Problem solving

Adapted Model of Occupational Self-Efficacy—Stage 3 Stage 3: Stage 3 will occur over a duration of 2 weeks								
Work-specific skills	The therapist will facilitate the activity and assess the participant's engagement in the activity.	- Assessment of specific components (i.e., range of motion, muscle strength, sensation, conation, and cognition) - Assessment of work abilities (i.e., work habits, work competence, and work endurance)	Stage 3: Creation of competency through occupational engagement • In this stage, the client continually improves so their view of themselves is shifted from a sick role (or I am unable to) to a more positive and independent role. • During this phase, the client is given the opportunity to equip themselves with the necessary skills in order to return to work. • This allows for the improvement of a positive sense of self-efficacy. This sense of independence could be measured by a functional independence measure. • The client will be able to engage in simulated tasks as per the requirement of the job description identified. • The client will be involved in work test placements for brief periods of time	60 min session(s)	Allowing the client to find ways to solve their own problems at work.	3 × 60 min sessions for 1 week	Individual therapy	Components to improve: - Endurance - Muscle strength - Memory - Sequencing - Problem solving - Instruction retention - Safety with tools
Curriculum vitae writing and professional behaviour	The therapist will facilitate basic training on how to construct a curriculum vitae and conduct oneself in a professional manner during job interviews.			60 mi session(s)	Encourage the client to reflect on the physical demands of the job and the possible workplace scenarios they might be faced with that could be challenging. Brainstorming practical solutions to problems.	5 × 60 min sessions for 1 week	Group therapy	Components: - Memory - Sequencing - Attention span - Concept formation - Problem solving - Social conduct - Time management - Coping skills

Adapted Model of Occupational Self-Efficacy—Stage 4 Stage 4: Stage 4 will occur over a duration of 1 week								
Coping skills and conflict management	The therapist will facilitate life skill groups.	- Assessment of work abilities (i.e., work habits, work competence, and work endurance)	Stage 4: The capable individual. • At this stage, the client has successfully engaged in work tasks that ultimately improve their volition and worker role. Their view of themselves improves as they succeed in work-related occupations. • During this phase, the client is placed into the open labour market or learnership facility This will provide the client with a sense of independence and allow them to feel a greater sense of self confidence and improve their self-efficacy levels.	60 min session	The client should be encouraged to discuss how they are coping within the workplace.	Once a week	Group therapy	Components to improve: - Self-confidence - Problem-solving skills - Reflection - Coping skills

**(a):** 

**Key informant**	**Age**	**Gender**	**Qualification**	**Years of experience**
P1 (rehab specialist)	25	Female	Bachelor's degree in occupational therapy	3
P2 (rehab specialist)	30	Female	Bachelor's degree in occupational therapy	8
P3 (rehab specialist)	32	Female	Bachelor's degree in occupational therapy	10
P4 (rehab specialist)	28	Female	Bachelor's degree in occupational therapy	6
P5 (rehab specialist)	30	Male	Bachelor's degree in physiotherapy	6

**(b):** 

**Participant**	**Age**	**Gender**	**Primary diagnosis description**	**Education**	**Pre-MI occupation**	**Classification of occupation**	**RTW after rehabilitation**
TB1 (TB survivor)	35	Female	PTB	Secondary	Teller	Light	Yes
TB2 (TB survivor)	53	Female	PTB	Secondary	Support group facilitator	Medium	Yes
TB3 (TB survivor)	54	Female	MDRTB	Tertiary	Nurse	Medium	Yes
TB4 (TB survivor)	40	Male	MDRTB	Secondary	Factory worker	Medium	Yes

*Note:* NB: classification of occupation. Light work is described as work where an individual lifts and carries no more than 10 kg occasionally. Medium work is described as work where an individual lifts and carries no more than 25 kg [19].

**Table 3:** Themes and categories.

Theme 1 Promoting a holistic model	Categories • Developing a client-specific program based on context • Therapist engagement in the program

Theme 2 The use of resources for activity engagement	Categories • Resources and staff in public health care • Lack of vocational rehabilitation assessment tools

## Data Availability

The data that support the findings of this study are available on request from the corresponding author, Dr. Mogammad Shaheed Soeker.
